# Supplementary material for: The interactome of intact mitochondria by cross-linking mass spectrometry provides evidence for coexisting respiratory supercomplexes
Source: Mol Cell Proteomics. 2017 Dec 8;17(2):216–32. doi: 10.1074/mcp.RA117.000470 (PMC5795388; doi:10.1074/mcp.RA117.000470)
Supplement: Supplemental Data [file supp_RA117.000470_133922_0_supp_23978_fzffwf.zip › spectra_annotation/mito_spectra_annotation/244-1-4-1-7-1.pdf]

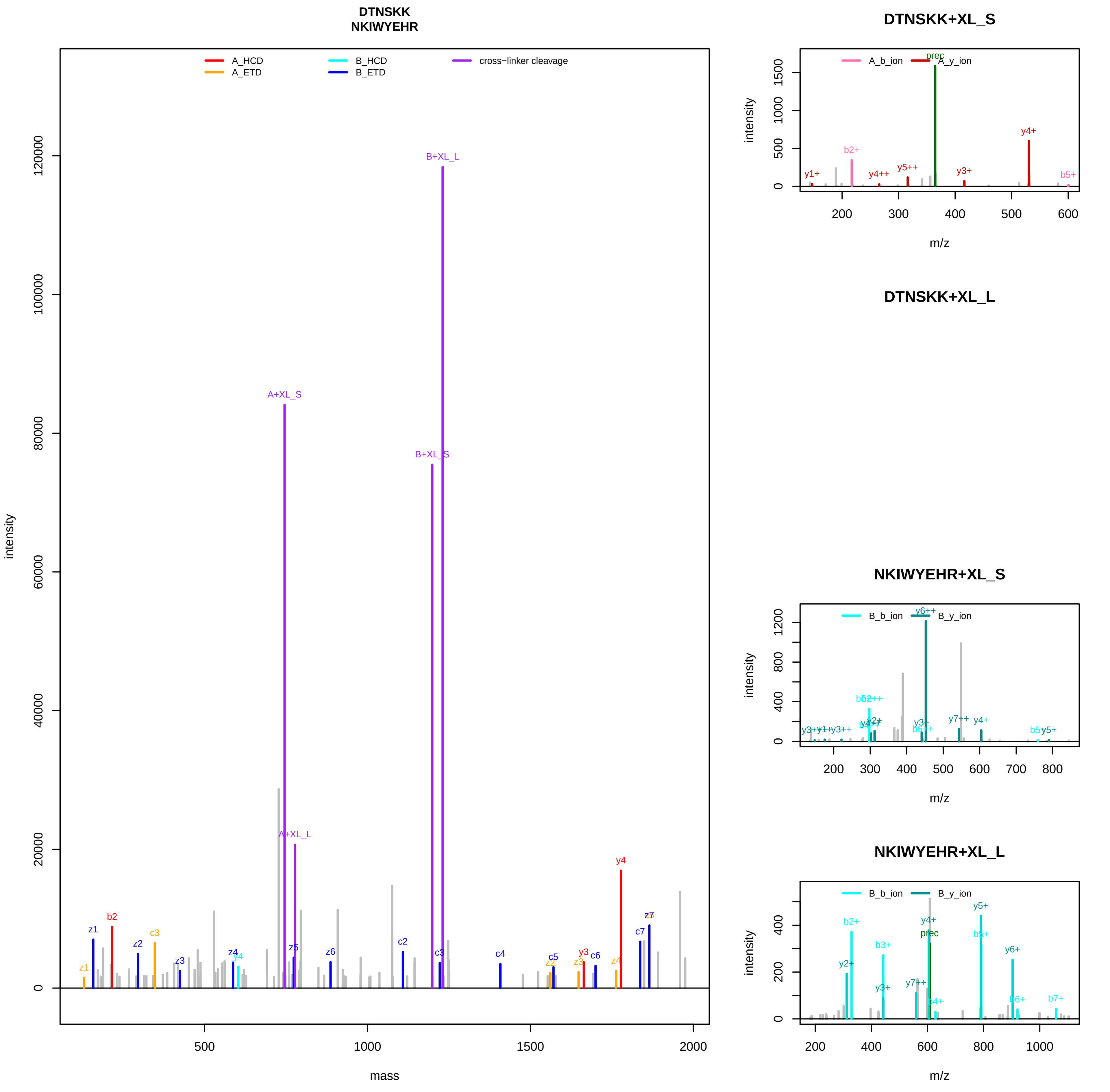
DTNSKK
NKIWYEHR
A_HCD
A_ETD
B_HCD
B_ETD
cross−linker cleavage
B+XL_L
A+XL_S
B+XL_S
A+XL_L
intensity
mass
0
20000
40000
60000
80000
100000
120000
500
1000
1500
2000
z1
b2
z2
c3
z3
z4
y4
z5
z6
c2
c3
c4
c5
z2
z3
y3
c6
z4
y4
c7
z7
DTNSKK+XL_S
A_b_ion
A_y_ion
prec
b2+
y1+
y4++
y5++
y3+
y4+
b5+
m/z
DTNSKK+XL_L
NKIWYEHR+XL_S
B_b_ion
B_y_ion
y6++
y7++
y4+
b5+
y5+
NKIWYEHR+XL_L
B_b_ion
B_y_ion
b2+
y2+
b3+
y3+
y7++
y4+
prec
y5+
b5+
y6+
b6+
b7+
b4+
